# Performance Analysis and Microbial Community Evolution of In Situ Biological Biogas Upgrading with Increasing H_2_/CO_2_ Ratio

**DOI:** 10.1155/2021/8894455

**Published:** 2021-02-09

**Authors:** Viola Corbellini, Cuijie Feng, Micol Bellucci, Arianna Catenacci, Tatiana Stella, Anna Espinoza-Tofalos, Francesca Malpei

**Affiliations:** ^1^Department of Civil and Environmental Engineering, DICA, Politecnico di Milano, Environmental Section, Piazza L. da Vinci 32, 20133 Milano, Italy; ^2^Department of Earth and Environmental Sciences, DISAT, University of Milano-Bicocca, Research Group of Environmental Microbiology, Piazza della Scienza, 1, 20126 Milano, Italy

## Abstract

The effect of the amount of hydrogen supplied for the *in situ* biological biogas upgrading was investigated by monitoring the process and evolution of the microbial community. Two parallel reactors, operated at 37°C for 211 days, were continuously fed with sewage sludge at a constant organic loading rate of 1.5 gCOD∙(L∙d)^−1^ and hydrogen (H_2_). The molar ratio of H_2_/CO_2_ was progressively increased from 0.5 : 1 to 7 : 1 to convert carbon dioxide (CO_2_) into biomethane via hydrogenotrophic methanogenesis. Changes in the biogas composition become statistically different above the stoichiometric H_2_/CO_2_ ratio (4 : 1). At a H_2_/CO_2_ ratio of 7 : 1, the methane content in the biogas reached 90%, without adversely affecting degradation of the organic matter. The possibility of selecting, adapting, and enriching the original biomass with target-oriented microorganisms able to biologically convert CO_2_ into methane was verified: high throughput sequencing of 16S rRNA gene revealed that hydrogenotrophic methanogens, belonging to *Methanolinea* and *Methanobacterium* genera, were dominant. Based on the outcomes of this study, further optimization and engineering of this process is feasible and needed as a means to boost energy recovery from sludge treatment.

## 1. Introduction

Global warming has been proven as the consequence of increased carbon concentration in the atmosphere resulting from greenhouse-gas emissions mainly of carbon dioxide (CO_2_) derived from human activities. According to the latest available data [1], China is the greatest producer of CO_2_ with 10.9 billion tons of equivalent CO_2_ released every year, followed by the USA (5.1 Gt per year) and the European Union (3.2 Gt per year). Among European countries (EU28), Italy is ranked 3rd (0.36 Gt) and 18th worldwide. Accordingly, the imposition of increasingly restrictive limits on municipal wastewater treatment plants (WWTPs), as well as the need to reduce the use of fossil sources, requires plants to become energy self-sufficient [2]. Energy is conventionally regained through biogas production from anaerobic digestion (AD). Biogas, mainly composed of 55-70% CH_4_, 30-45% of CO_2_, and other trace gases (nitrogen, oxygen, water, hydrocarbons, ammonia, and siloxanes) [3], can be utilized in combined heat and power engines or, after removal of CO_2_ (biogas upgrading) and other impurities, as biomethane. In this last case, the market already offers various chemical/physical upgrading technologies. However, one main drawback lies in simply splitting CO_2_ from the biogas flux. Nonetheless, given the considerable challenges in terms of energy/chemical consumption, researchers are investigating alternative solutions [4]. Among these, the biological CO_2_ conversion into methane (CH_4_) (Equation (1)) is attractive.

(1) $$\begin{array}{c} 4{\text{H}}_{2}+{\text{CO}}_{2}⟶{\text{CH}}_{4}+2{\text{H}}_{2}\text{O}∆{\text{G}}_{0}=-135.6\text{ kJ}∙{\text{mol}}^{-1}. \end{array}$$

It allows the simultaneous reduction of CO_2_ and increase of CH_4_ yields, towards a more sustainable biogas upgrading technology which converts biogenic CO_2_ into an energy source, with a negative carbon emission footprint in terms of fossil CO_2_ [5]. The high biological upgrading requirement of exogenous H_2_ to allow CO_2_ conversion can be satisfied by exploiting the excess off-peak energy of naturally fluctuating renewables (wind, solar) to sustain the water electrolysis process as power to gas (P2G) [6]. In addition, in a WWTP equipped with sludge AD treatment, the biological biogas upgrading introduces two additional advantages: (i) the O_2_ coproduced along with H_2_ by water electrolysis could be used in the activated sludge treatment, which requires around 50% of the total WWTP energy needs [2]; (ii) the effluent water from a WWTP could be used as source water for electrolysis, although pretreatment may be needed [7].

The biological CO_2_ methanization process is carried out by archaea belonging to the orders of *Methanobacteriales*, *Methanomicrobiales*, and *Methanococcales*, which are classified as hydrogenotrophic methanogens [8]. Hydrogenotrophic methanogens are commonly found in every anaerobic digester playing a significant role in scavenging H_2_ in order to maintain a low partial pressure (pH_2_ < 10 Pa).

So far, three main applications of this novel upgrading technology were studied at the lab-scale: *in situ*, *ex situ* [9–11], and *hybrid* [12]. In the *in situ*, H_2_ is fed into a biogas reactor where it is used with the CO_2_ produced by organic substrate degradation. The main drawbacks of the *in situ* system are: (a) accumulation of intermediates due to the increased H_2_ partial pressure (pH_2_) [13], (b) increase of pH due to a progressive depletion of endogenous dissolved CO_2_ [9, 12, 14], and (c) low hydrogen solubility which can limit a homogeneous and efficient distribution of the gas in the liquid phase [13, 15–18].

Regarding the effect on the organic degradation chain, Corbellini et al. [21] tested a novel approach, performed in semibatch bioreactors, consisting in progressively increasing H_2_ dosage in order to acclimate and develop a specialized biomass. Few other studies can be found in the literature pertaining *in situ* biogas upgrading treating sewage sludge and specifically focused on the H_2_/CO_2_ ratio for process optimization [19, 20]. Indeed, it is crucial to obtain a deeper knowledge of the effect of the H_2_/CO_2_ ratio in shaping a consortium of bacteria and archaea able to steadily and simultaneously maximize CO_2_ methanization and degrade the organic substrates.

In this study, the biological *in situ* biogas upgrading was operated in two lab-scale continuous stirred tank reactors (CSTR) working in parallel in order to evaluate the repeatability of the process and of the experimental outcomes: this latter aspect is of importance as it is seldom addressed when testing biological biogas upgrading, and the vast majority of literature reports on single operating reactors. The experimental plan was determined with two main purposes: to investigate the effect of the H_2_/CO_2_ ratio on the efficiency of biogas upgrading and on the stability of the process and to study the evolution of the specific microbiome in connection with the development of the process at the macroscale. Based on results of this investigation, further knowledge has been gained pertaining to (1) the relationship between the biogas rate produced and the main operational parameters; (2) the H_2_/CO_2_ ratio up to which the system can be pushed before encountering instability; (3) the effects of increased H_2_/CO_2_ ratio on the microbial community, which was analysed at different experimental stages; and (4) the repeatability of the process.

## 2. Materials and Methods

### 2.1. Experimental Setup and Design

Two identical lab-scale CSTRs (total volume, *V*_tot_ = 16 L; working height, *H*_*w*_ = 31.3 cm; diameter, *D* = 30 cm; working volume, *V*_*w*_ = 11 L), hereafter referred to as R1 and R2, were operated in parallel and daily fed with 0.5 L of a primary and secondary sludge mixture. The organic loading rate referred to the sludge (OLR_SL_) was 1.5 ± 0.1 gCOD∙(L·d)^−1^ and the hydraulic retention time (HRT) 22 days. The two reactors (Umwelt GmbH) were equipped with peristaltic pumps for sludge loading and discharging and for controlling the pH at 7.4 ± 0.2 by adding acid or basic solutions (0.5 M HCl, 1 M NaOH). H_2_ was injected in each reactor with an additional peristaltic pump (Velp Scientifica, Type SP-311/2, Italy) through an aluminium tube (Ø = 6 mm) sunk at 3/4 of the total sludge height.

Vigorous mixing at 120 rpm was assured by vertical shaft stirrers. External heating jackets maintained the internal temperature at 36.7 ± 1°C. The biogas flow was quantified by a gas meter (RITTER Apparatebau GmbH & Co. KG) and analysed online with a gas analyser (AwiFLEX Cool+, Awite Bioenergie GmbH) for CO_2_ (range 0-100%), CH_4_ (range 0-100%), and O_2_ content (range 0-25%) by pressure and infrared compensation methods, while the H_2_S (up to 1500 ppm) was measured by an electrochemical sensor. Hydrogen was analysed, twice per week, using gas chromatography (DANI Master GC) coupled with a flame ionization detector (FID Nukol fused silica). Reactors were operated for a total of 211 days, divided into VIII Periods (Table 1). In Period I (start-up period), both reactors were fed on the sewage sludge mixture only. Then, while maintaining a constant OLR_SL_, H_2_ was progressively fed to reactors at increasing H_2_/CO_2_ ratios: periods from II to VI were dedicated to the enrichment (H_2_/CO_2_ from 0.5 up to 4); in Periods VII and VIII, H_2_/CO_2_ ratios above the stoichiometric value, and equal to 6 and 7, were adopted. Table 1 reports other relevant operating conditions for each period, since the OLR_tot_ is the sum of contributions given by the sewage sludge mixture (OLR_SL_) and the H_2_, both computed on COD basis (conversion factor 8 gCOD∙gH_2_^−1^). The length of periods up to the stoichiometric H_2_/CO_2_ ratio was assumed as in Corbellini et al. [21], where such durations were defined in order to simultaneously achieve a stable response to a perturbation and shorten the start-up time required to adapt the biomass to an increasing H_2_ supply.

The amount of H_2_ daily dosed in each reactor during Period *i* (Dose(H_2_)_period_*i*_) was calculated based on the H_2_/CO_2_ ratio and on the average rate of CO_2_ produced during the (*i*-1)^th^ Period, according to [21]. The daily H_2_ amount was supplied by activating the H_2_ pump for 20 pulse/day and adjusting the pump speed in order to reach the H_2_ amount for each experimental period.

### 2.2. Inoculum and Feedstock Preparation and Characteristics

Both the feeding sludge and the inoculum were taken from a full-scale municipal WWTP located in Bresso (Milan Area, Italy). The feeding sludge (0.5 L∙d^−1^) is a mixture of primary and waste activated sludge (WAS), directly taken from the primary settling tank where WAS is recirculated. The inoculum (10 L) was collected from the mesophilic full-scale digester. The sludge mixture was hashed and sieved (2 mm) to prevent clogging of the pump tubes; then, it was stored at -20°C and used after thawing. The main characteristics of the substrates are reported in Table 2. Two biochemical methane potential tests (BMP) were performed on the sludge mixture at the beginning and at the end of the experimentation, in this last case using the reactor effluent as inoculum.

### 2.3. Monitoring Strategy

Total and volatile solids and total COD were measured every 10 days on the feeding sludge. Total and volatile solids, volatile fatty acids, soluble COD, and alkalinity of the effluent digestate were determined twice a week. Based on the results, two indexes were calculated for each experimental period: (i) the amount of H_2_ consumed (*H*_2, eff_), according to [21], and (ii) the percentage of volatile solids removed in order to monitor the effect of H_2_ injection on the organic substrate degradation, according to Equation (2).

(2) $$\begin{array}{c} \text{VS }\left(\%\right)=\frac{{\text{VS}}_{\text{in}}-\text{V}{\text{S}}_{\text{out}}}{{\text{VS}}_{\text{in}}}\cdot 100, \end{array}$$

where VS_in_ and VS_out_ (gVS∙L^−1^) are the volatile solid concentrations in the influent and effluent sludge.

### 2.4. Analytical Methods

Total and volatile solids (TS, VS) were measured according to Standard Methods 2540 [22]. Alkalinity was measured by titration with H_2_SO_4_ up to pH 4.3, using an automatic titrator (Hach Lange BIOGAS Titration Manager, USA). Soluble COD (sCOD) was measured using spectrophotometric test kits (DR6000 UV-VIS with RFID by Hach-Lange) after filtration (0.45 *μ*m). Volatile fatty acid (VFA, acetic, propionic, isobutyric, butyric, isovaleric, and valeric) concentrations were determined according to Standard Methods 5560 [22], using a gas chromatograph (DANI Master GC) coupled with a flame ionization detector. Hereafter, the term TVFA indicates the total concentrations of VFA expressed as equivalent COD. Total COD was determined according to Standard Methods 5220 [22]. Biogas composition (CO_2_, CH_4,_ H_2_, O_2_, and N_2_) was characterized twice a week using gas chromatography (DANI Master GC Analyser equipped with two columns HayeSep Q and Molesieve 5A). The Automatic Methane Potential Test System II (AMPTS II, Bioprocess Control®) was used for BMP determinations. Tests were performed in duplicate, at mesophilic conditions (35 ± 0.5°C) and adopting a substrate to inoculum (S/I) ratio of 0.5 on VS base according to the Italian BMP standard method [23].

### 2.5. Statistical Analysis

Statistical analyses were carried out using the SPSS v.25 software in order to assess: (i) the repeatability in the operation of the two reactors, R1 and R2, and (ii) the significance of the differences observed between the eight experimental periods. Data distributions were firstly verified, both graphically (results not shown) and numerically. The Kolmogorov-Smirnov and Shapiro-Wilk tests (significance level = 0.05) were used to test variables against normality. As normality conditions were not always satisfied, the nonparametric Mann–Whitney *U* test and Kruskal-Wallis test (significance level = 0.05) were used to compare the selected dependent variables to the independent categorical variables of interest (reactors or periods).

### 2.6. Sampling, Amplification of 16S rRNA Gene, Sequencing, and Sequence Analyses

**Figure 1:**
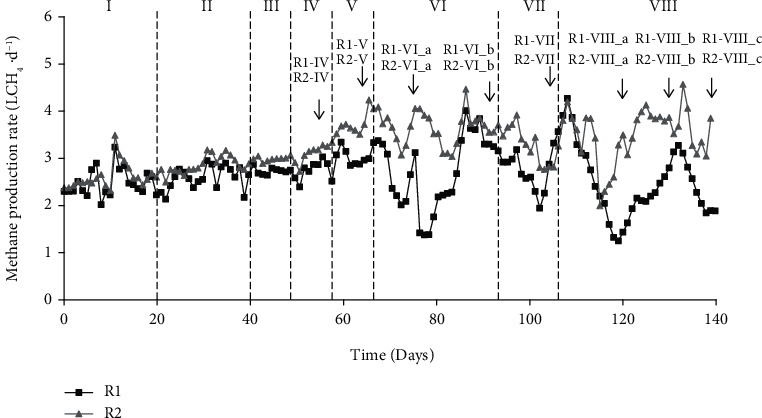
Methane rate produced by R1 and R2 reactors during the experiment; arrows indicate DNA extraction sampling points: labels include the name of the reactor sampled (R1 or R2), the experimental period in roman letters, and when needed, a letter to distinguish samples from the same reactor and period (a, b, or c).

A total of 16 samples were taken from R1 and R2 reactors for high throughput 16S rRNA gene sequencing for microbial community analyses. Sampling points were selected according to the following scheme: one sample was collected at the end of Periods IV (R1-IV and R2-IV) and V (R1-V and R2-V), two during Period VI (R1-VI and R2-VI), one at the end of Period VII (R1-VII and R2-VII), and three in the last Period VIII (R1-VIII_a; R1-VIII _b; R1-VIII _c; R2-VIII _a; R2-VIII _b; R2-VIII _c), as summarized in Figure 1.

Samples were centrifuged (7000 rpm, at 4°C for 10 min) to obtain around 2 g of cell pellet. The total microbial DNA was extracted using FastDNA Spin for Soil kit (MP Biomedicals, Solon, USA) according to the manufacturer's protocol. The bacterial V5-V6 hypervariable regions of the 16S rRNA gene were PCR-amplified using 783F and 1046R primers [24, 25], while for the archaeal communities a fragment of the 16S rRNA gene was PCR-amplified using the IA_349F-IA_571R primers [26]. The multiplexed libraries were prepared using a dual PCR amplification protocol. The bacterial PCR was performed in 2 × 50 *μ*L volume reactions with GoTaq® Green Master Mix (Promega Corporation, Madison, WI) and 1 *μ*M of each primer, and the cycling conditions were initial denaturation at 98°C for 30 s; 20 cycles at 98°C for 10 s, 47°C for 30 s, and 72°C for 5 s; and a final extension at 72°C for 2 min. The archaeal PCR was performed in 4 × 25 *μ*L volume reactions with Phusion high fidelity polymerase (Thermo Scientific) and 2 *μ*M of each primer, and the cycling conditions were initial denaturation at 96°C for 4 min; 10 cycles at 96°C for 30 s, 68°C for 30 s, and 72°C for 25 s; then 30 cycles at 96°C for 30s, 58°C for 30 s, and 72°C for 25 s; and a final extension at 72°C for 5 min. Amplicons were purified with the Wizard® SV Gel and PCR Clean-up System (Promega Corporation, Madison, WI, USA) according to the manufacturer's instructions. After the purification, DNA quality was evaluated spectrophotometrically, and DNA was quantified using Qubit® (Life Technologies, Carlsbad, CA). The Illumina Miseq sequencing was carried out at Consorzio per il Centro di Biomedicina Molecolare (Trieste, Italy). Reads from sequencing were de-multiplexed according to the indexes and then quality filtered. Quality-filtered reads were assembled into error-corrected amplicon sequence variants (ASVs) using DADA2 v1.4.0 [27], which represent unique bacterial/archaeal taxa. Assembled ASVs were assigned taxonomy (phylum to species) using the Ribosomal Database Project (RDP).

Rarefaction curves were performed using the PAST3 software. Heat maps were produced with the STAMP software. Nonmetric multidimensional scaling (NMDS) analyses based on Bray-Curtis dissimilarity index were conducted using the vegan packages of R (R version 3.6.0). The discussion of the data is focused on the most abundant bacterial and archaeal genera in the community with relative abundance of at least 0.5% for archaea and >1% for bacteria.

## 3. Results and Discussion

### 3.1. Repeatability between Reactor Operation

The repeatability between reactors was statistically tested to assess whether it was reliable to group output data. For this purpose, 5 variables were used: three “gas phase” variables, biogas rate, and its composition (CH_4_ and CO_2_) and two “liquid phase” variables, alkalinity, and TVFA. They were evaluated either pooling all the data together, independently on the period (case A) or separating testing variables period by period (case B). As for case A, variability in data distributions was found between R1 and R2 if considering biogas rate (*U* = 15′727; asymptotic significance, ASig < 0.001; mean ranks, MRk: R1 = 103; R2 = 182). Results turned out opposite when testing CH_4_ (*U* = 1′009, ASig. = 0.097, MRk: R1 = 55.3; R2 = 45.7) and CO_2_ contents (*U* = 1′416, ASig. = 0.252, MRk: R1 = 47.2; R2 = 53.8) as well as alkalinity (*U* = 1′148, ASig. = 0.054, MRk: R1 = 38.3; R2 = 48.7) and TVFA (*U* = 387, ASig. = 0.352, MRk: R1 = 32.6; R2 = 28.4). Although the conditions inside R1 and R2 were found statistically similar, different distributions of data were observed in the biogas rate but displaying statistically comparable gas compositions. This is probably to be ascribed not only to the different microbial communities that developed between the two reactors (see par. 3.5) but also to specific local environmental conditions. After a certain period of H_2_ dosing, bioreactors might have improved or conversely limited particular degrading pathways or secretion of specific enzymes. This aspect needs to be further investigated, by adopting a target-oriented experimental plan focused on this purpose. The period by period comparison (case B) between reactors provided more accurate results (see Table S1 in the Supplementary Material): different biogas rate distributions between reactors were observed for all eight periods with significance values well below the 0.05. Conversely, CO_2_ and CH_4_ content distributions were the same for R1 and R2 during all periods, except Periods I (exact significance, Sig. = 0.021) and VI (Sig. = 0.008). Results on alkalinity and TVFA confirm what observed testing the entire set of data, from Period I to Period VIII: the chemical conditions inside R1 and R2 were always statistically comparable, except for Periods VI (Sig. = 0.004) and VIII (Sig. = 0.004). It should be noted that Period VI corresponds to the stoichiometric dosage of H_2_: starting from this period, significant variability is expected. Based on this statistical analysis, all results obtained are presented and discussed separately for the two reactors, especially with reference to the biogas rate.

### 3.2. Statistical Significance of Biogas Upgrading

The three “gas phase” variables were statistically tested using the nonparametric, Kruskal-Wallis test to verify the significance of the observed differences between the eight periods (see Table S2 in the Supplementary Material). Significance values were found below 0.001 for all the three variables, then providing strong evidence of a difference between the mean ranks of at least one pair of periods. Following the rejection of Kruskal-Wallis tests, post hoc procedure for pairwise multiple comparisons was performed to identify which pairs were different. Dunn's pairwise tests were carried out using the Bonferroni correction to adjust the rejection level on the total number of tests (results are summarized in Table S3, Supplementary Materials). This test revealed that, for both reactors, the biogas rate produced during Period I becomes statistically different starting from Period V when approaching the stoichiometric dosage of H_2_; then, a different behaviour between R1 and R2 was observed: as for R1, the biogas rate produced during Periods VII (Sig. = 0.109) and VIII (Sig. = 0.878) returned being statistically the same as what was produced during Period I. Conversely, the biogas rates produced during Periods from V to VIII in R2 present strong evidence (Sig.<0.026) of being statistically different from those produced in both Periods I and II. Similar results were observed for CO_2_ and CH_4_ content, instead: changes in biogas composition become statistically different from Period II, starting from Period VI (Sig.<0.043); increasing the H_2_/CO_2_ ratio above the stoichiometric, however, did not change significantly, from a statistical point of view, the biogas composition.

### 3.3. Reactor's Performance

#### 3.3.1. Period I: Pre-H_2_ Phase

As shown in Figure S1 in Supplementary Materials, during Period I, initial variability of methane yields was observed in both reactors. However, after 97 days, corresponding to three HRTs, the steady-state was reached in both reactors, and methane yields of 0.26 and 0.28 NLCH_4_∙gVS^−1^ for R1 and R2, respectively, resulted being comparable to the BMP values (0.229 ± 0.001 and 0.210 ± 0.002 NLCH_4_∙gVS^−1^) measured at the beginning and at the end of the experiment.  Table 3 reports the average gaseous flows of methane, carbon dioxide, and hydrogen throughout the periods of the experiment for the two parallel reactors. Since the beginning, higher CO_2_ and CH_4_ production rates are observed in reactor R2, compared to R1, although similar biogas composition is detected. Such deviation in the biogas rate between R1 and R2 increases during the experiment, despite the same feeding being maintained during all periods: this is probably due to small unintentional changes in the environmental conditions of the two reactors which could have led to different speciation in the microbial community (i.e., alkalinity, nutrients, and VFA, which could, for example, determine the secretion of different specific enzymes). Under normal conditions, such variations result in negligible effects, such as the slightly higher concentrations of unconverted VFA found in R1 up to phase IV. Thereafter, starting to stress the system with H_2_ dosing, it is possible that the process turned out to be more sensitive even to small variations, then resulting in more pronounced differences between reactors (see alkalinity and VFA concentrations starting from phase V, when approaching the stoichiometric H_2_/CO_2_ ratio), which led to the diverse rates of biogas produced reported in Table 3.

**Figure 2:**
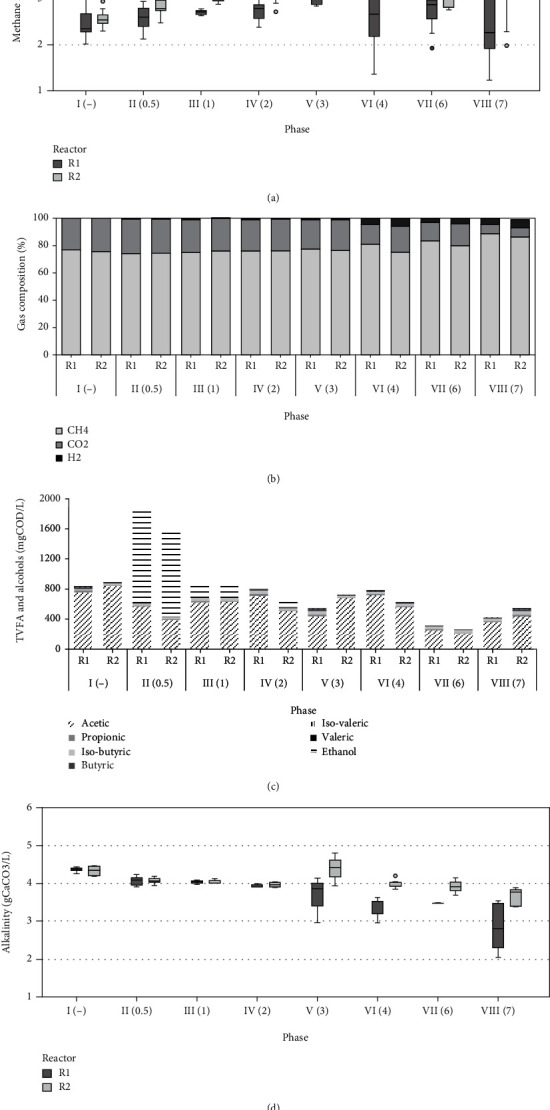
Mean values of parameters measured during each of the eight experimental periods (in brackets the H_2_/CO_2_ ratio): (a) box plots of methane rate (circles indicate outliers); (b) biogas composition; (c) TVFA composition and alcohols; (d) box plots of alkalinity concentrations (circles indicate outliers).

Figure 2 shows, for both reactors, mean values of parameters monitored for each period.

Reactors behaved similarly during Period I, with high methane (76.2%) and low CO_2_ (23.8%) contents in the biogas (Figure 2(b)) and comparable alkalinity (Figure 2(c)) and TVFA concentrations (Figure 2(d)), with the acetic acid prevailing.

#### 3.3.2. Periods II-VI: Enrichment of the Hydrogenotrophic Methanogens

During Periods II to VI (days 21-94), H_2_ was injected by progressively incrementing the dosage. Since very low concentrations were detected in the output gas, it can be concluded that H_2_ was completely consumed by the process, while observing a slight increase in the produced methane rate as shown in Figure 2(b). This confirmed that hydrogenotrophic methanogens usually work below their H_2_ rate consumption capacity [28], then being able to consume more hydrogen available as soon as they come into contact with it. Furthermore, the anaerobic degradation of the substrate was not affected, and VFAs were easily consumed during the process as no accumulation was observed: biogas production was stable as well as volatile solids degradation, as reported in Table 4. However, as shown in Figure 2(c), ethanol peaks of 3.2 and 1.4 gCOD∙L^−1^ in reactors R1 and R2 were reported during Phase II, immediately in response to H_2_ injection. To the best of the authors' knowledge, in the recent literature on *in situ* biogas upgrading, ethanol accumulation has not yet been reported. It can probably be ascribed to the regulatory role of H_2_ in the normal operation of anaerobic digesters. Ethanol is formed from sugars during the acidogenesis step [29], and it is generally oxidized by syntrophic bacteria and methanogens [30, 31]. Low H_2_ concentrations allow thermodynamic degradation of alcohols and fatty acids by H_2_-producing syntrophic bacteria [30]; thus, the amount of extra H_2_ provided to a biomass which is not yet properly acclimatized influenced alcohol oxidation, possibly leading to the ethanol accumulation. Nevertheless, although the H_2_ supply was not interrupted, ethanol decreased to 0.2 gCOD∙L^−1^ in both reactors during Period III (H_2_/CO_2_ 1 : 1) and was almost completely depleted in Period IV. This is probably due to an increased activity of H_2_-scavenging microorganisms, which have promptly reduced the exogenous H_2_, then allowing for alcohol degradation.

Furthermore, it is generally reported that around 40% of the total H_2_ provided is utilized via homoacetogenesis plus acetoclastic methanogenesis pathways [32]. Despite this, in this study, the acetate concentrations registered were stable in both reactors (0.6 gCOD∙L^−1^ in R1 and 0.4 gCOD∙L^−1^ in R2), which appears to be in contrast with this significant H_2_ consumption route.

When reaching the stoichiometric H_2_/CO_2_ (4 : 1) value in Period VI, high variability in the biogas yield was observed in R1, with a methane production rate varying from 1.5 up to 4 NLCH_4_∙d^−1^ (Figure 2(a)). Regardless of the unstable biogas rate, CO_2_ content decreased to 14% while CH_4_ rose to 81%. R2 behaved differently, having restrained variations of biogas and methane rates (3 up to 4.46 NLCH_4_∙d^−1^) but with a lower reduction of CO_2_ content (from 23 to 19%) and CH_4_ increase (75%) (Figure 2(b)). The authors believe that the lower production of biogas in R1 led to a higher amount of H_2_ available for a lower quantity of CO_2_ produced, thus allowing for higher CO_2_ conversion.

#### 3.3.3. Periods VI-VIII: Overstoichiometric Assessment

In Periods VII and VIII, both reactors were operated at H_2_/CO_2_ ratio of 6 : 1 and 7 : 1. In the first overstoichiometric period, CO_2_ was further converted in order to achieve a CH_4_ content of 84% and 80% in R1 and R2, respectively. VFAs were very low, indicating a stable process. When the H_2_/CO_2_ ratio was further increased to 7 : 1, both R1 and R2 showed a maximum methane production rate of 4 and 4.5 NLCH_4_∙d^−1^, even though R1 displayed high variability. Furthermore, VFAs and ethanol were almost constant. In this final period, R1 reached the lowest CO_2_ content of 4.5% and the maximum CH_4_ content of 90.3% (Figure 2(b)). The organic substrate degradation was not negatively affected by exogenous H_2_ injections. Indeed, a BMP test on the sludge mixture was carried out (day 137), using as inoculum a mixture of digestates taken from the two reactors. This resulted in 210 ± 1.5 NmLCH_4_∙gVS^−1^, a value comparable to 229 ± 1 NmLCH_4_∙gVS^−1^ obtained at the beginning of the test using the digestate from the WWTP Bresso as inoculum.

#### 3.3.4. Comparison with Literature

Table 5 summarizes several studies on the *in situ* biogas upgrading by H_2_ injection at different operational conditions. It can be seen that few studies pertaining to the *in situ* biogas upgrading process performed at mesophilic conditions with sewage sludge allow a direct comparison with the present work. Wang et al. [33] utilized Synthetic Coke Oven Gas (SCOG 92% H_2_ and 8% CO), as hydrogen source injected, with a hollow fibre membrane (HFM) module, into a CSTR applying the stoichiometric biomethanation ratio (H_2_/CO_2_ = 4 : 1). Biogas was completely upgraded (98-99 CH_4_%) only when pH value was fixed at 8. Agneessens et al. [19] tested pulse H_2_ injections in batch mode with a H_2_/CO_2_ ratio varied from 2 : 1 to 10 : 1. The ratio 8 : 1 turned out to be the best choice with a final CO_2_ content of 11.8%. Later on, in a study performed by Corbellini et al. [21], the H_2_/CO_2_ ratio was increased from 1 : 1 to a maximum of 4 : 1 in a semicontinuous mode, but the CH_4_ fraction reached in the biogas was a maximum 80%. The present study investigated the process with higher volumes (16 L) than all previous studies where the volume adopted was always lower than 3.5 L [49]. The H_2_ injection was tested with in a wider range of H_2_/CO_2_ ratios and in CSTR bioreactors. Moreover, the repeatability of the process was evaluated with two parallel operating reactors, a topic that has never been addressed previously. The effects of the aforementioned aspects on biological H_2_ and CO_2_ methanization behaviour are of great importance for practical application with a view towards larger-scale studies.

### 3.4. Alkalinity Trends over CO_2_%

Mean values of alkalinity concentrations in both reactors are reported in Figure 2(d). From Period II, alkalinity was consumed in both reactors along with the progressive increase of the H_2_/CO_2_ ratio. This evidence is directly related to CO_2_ consumption in the liquid phase, in accordance with a previous study [33]. The overall CO_2_ reduction, at the end of the experiment, was significantly higher in R1 (-40%) than in R2 (-14%) (Table 4), also confirmed by the lower CO_2_ content registered in the output biogas. The buffer capacity of anaerobic digesters is crucial to maintain neutral and stable pH values. For this reason, full-scale applications of the *in situ* upgrading process are limited if the influent organic substrate is not sufficient to restore alkalinity.

### 3.5. High Throughput 16S rRNA Gene Amplicon Analysis

**Figure 3:**
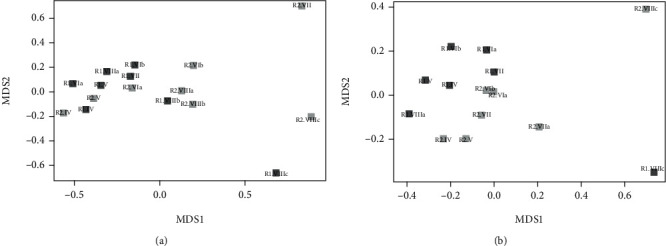
Nonmetric multidimensional scaling plots based on Bray–Curtis distances of the bacterial (a) and archaeal (b) communities at ASV level in R1 (light grey) and in R2 (dark grey).

As shown in Table S4 in Supplementary Materials, a total of 514,295 (bacteria) and 495,983 (archaea) reads were obtained from the 16 samples. All of them, except the archaeal R1-VIII_b and R2-VIII_b, reached a plateau, indicating that the number of ASVs covered the sample richness (Figure S2 in Supplementary Materials). Given not satisfactory archaea sequencing results of R1_VIII_b and R2-VIII_b (only around 200 reads and 5 ASV), these samples were not included for the further analyses. The evolution of the microbial communities over time and with respect to the digesters was performed by NMDS analysis (Figure 3).

For bacteria, distinct clusters could not be observed, while samples collected from the two reactors during the same period are closed to each other suggesting an evolution of the community due to the different operational parameters applied in the different experimental periods. On the contrary, for archaea, two clusters, which contain most of the samples of R1 and R2, could be identified. A neat shift of the community occurred at the end of the operational time because R1_VIIIc and R2_VIIIc are completely separated from the rest of the samples.

#### 3.5.1. Bacterial Community Composition

**Figure 4:**
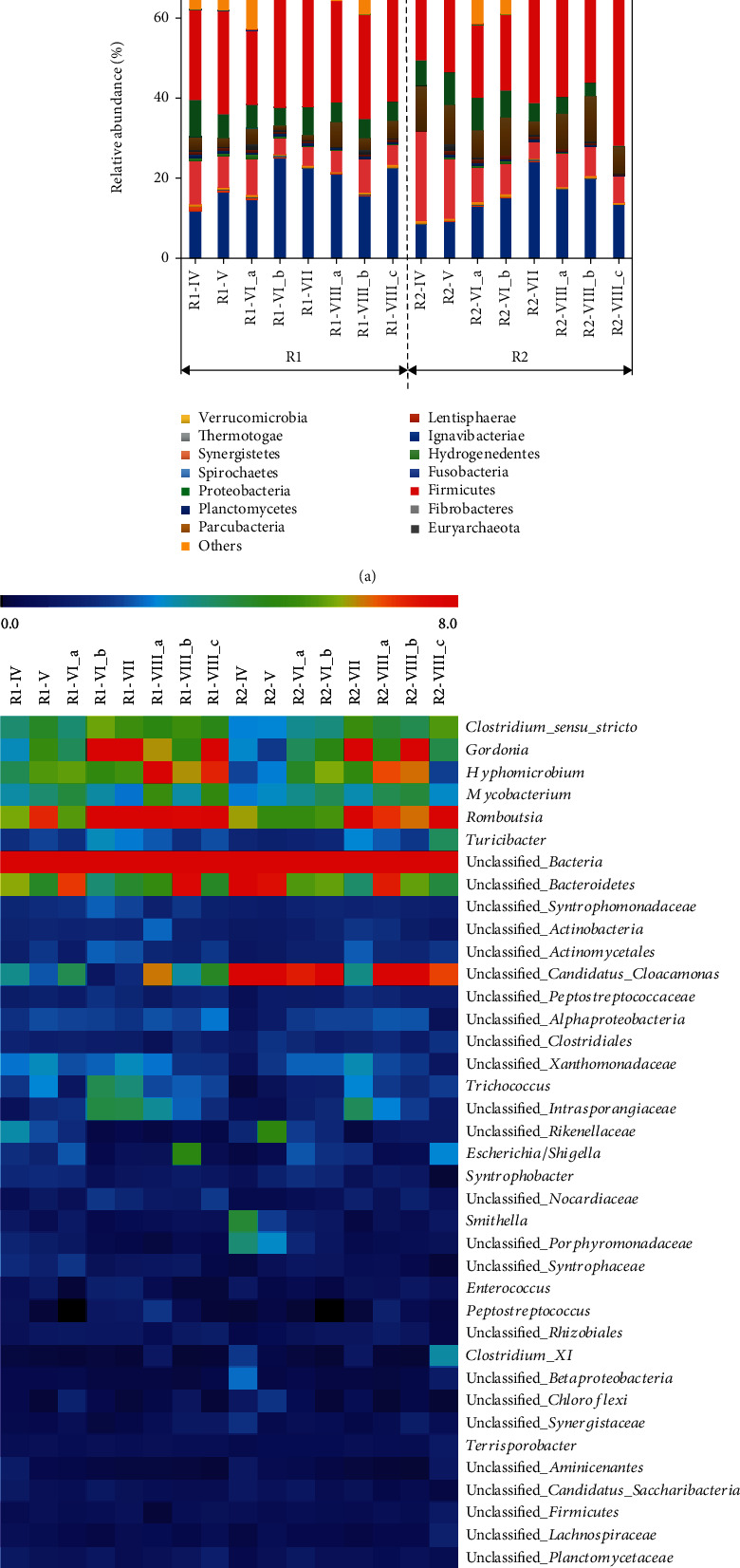
(a) Relative abundance of the bacterial phyla of two parallel reactors at several sampling points. (b) Heat maps of relative abundance (>1%) of the most abundant bacterial ASVs of R1 and R2. The scale limit starts from 0 (abundance equal to 1% because of the selection) to 8%. Increasing red colour indicates a higher value of relative species abundance, and blue colour indicates a lower relative abundance.

The bacterial community consisted of Firmicutes (17%-50%), Proteobacteria (12%-26%), Actinobacteria (9%-25%), and Bacteroidetes (4%-22%), while other phyla, such as Unclassified_bacteria (8%-15%), Cloacimonetes (1% -11%), and Synergistetes (1%-2%), were less abundant (Figure 4(a)). Relative abundances of the bacteria at the genus level are shown in Figure S3 of the Supplementary Materials. As in previous studies, the dominance of Firmicutes and Bacteroidetes in digesters is frequently observed [34], as they are hydrolytic bacteria [33, 35, 36] responsible for the breakdown of polymeric substrates, such as proteins, lipids, and polysaccharides. Proteobacteria, known as acidogenic bacteria, was one of the dominant phyla in this study, in line with previous studies in AD [36, 37]. It can be seen that the bacteria related to hydrolysis and acidogenesis processes constitute a large proportion, over 60%, of total bacteria [38]. Their proportion rose with the increase in the H_2_/CO_2_ ratio suggesting that increasing H_2_ dosage promoted the development of a microbial consortia enriched with hydrolytic and acidogenic bacteria, which enhanced the methane yield and purity. All further discussions on microbial analysis results were focused only on the most abundant ASVs with a relative abundance > 1%. 38 ASVs represent the most abundant members in all the samples. The evolution of the most abundant genera detected in the two reactors is represented as heat map at a genus level in Figure 4(b).

*Unclassified_Bacteroidetes Romboutsia*, *Unclassified_Candidatus_Cloacamonas*, *Hyphomicrobium*, and *Mycobacterium* were found to be the predominant genera in both reactors*. Unclassified_Bacteroidetes*, known to be involved in the polysaccharide and protein hydrolysis step during the AD process [39], were constantly present with an average of 7% in both reactors during all experimental periods. *Romboutsia*, known as homoacetogens [40], increased its relative abundance constantly from 5% to 9% during the experiment. However, the recorded increases of *Romboutsia* do not seem to be confirmed by the low levels of acetate found during the experimental trial. This is probably due to the faster acetate consumption dynamics of acetoclasts in forming methane, compared to the acetate sampling and measurement intervals. Another most abundant bacterial genus was *Hyphomicrobium*, which increased in both reactors from 3.9% to 7.1% in R1 and 2.1% to 6.4% in R2. Bacteria belonging to this genus are known to cooperate with methanogens (i.e., *Methanosarcina*) in simultaneous denitrification and methanogenesis [41] and the aforementioned *Romboutsia* (from 5.8% up to 8.7% relative abundance) indicating a constant H_2_ consumption also by homoacetogenesis. Also, members belonging to *Gordonia* increased in both reactors (from 3% up to 9% in R1and 3% to 8% in R2). These bacteria are known to be able to degrade environmental pollutants such as polycyclic aromatic hydrocarbons [42] that are normally present in wastewater activated sludge [43].

#### 3.5.2. Archaeal Community Composition

**Figure 5:**
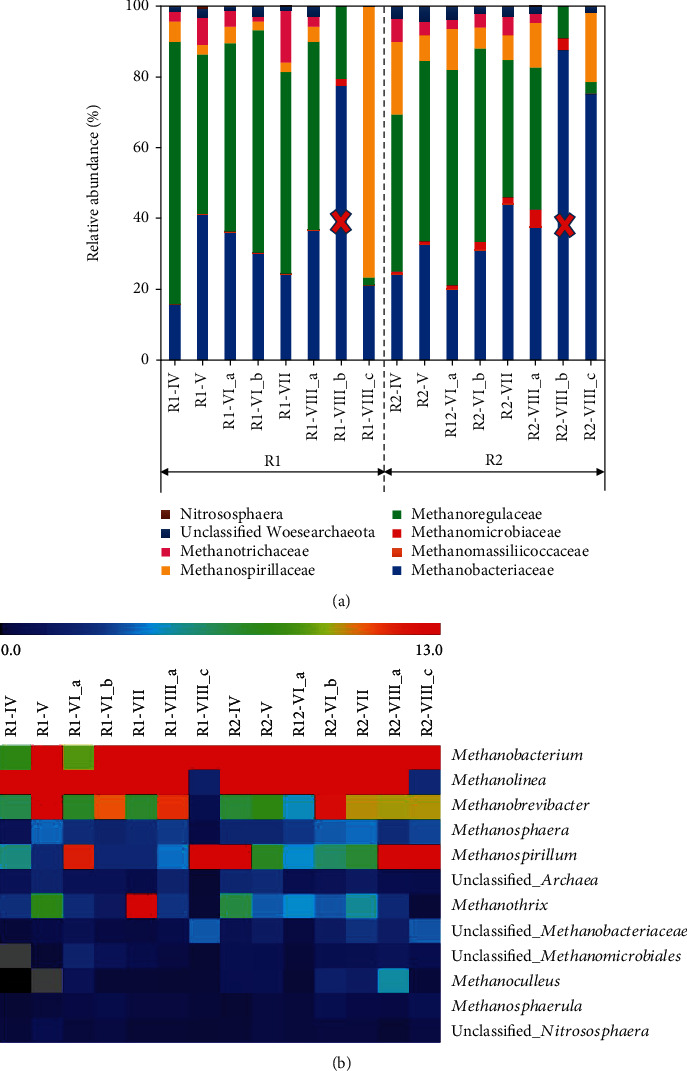
(a) Relative abundance of the archaeal community structure on the family level. R1_III b and R2_III b labelled with “X” are without consideration due to unideal archaea sequencing results. (b) Heat maps of relative abundance (>0.5%) of the most abundant archaeal ASVs of R1 and R2.

In the archaeal community patterns, two phyla, Euryarchaeota (98%) and Woesearchaeota (1.5%), and 8 families were identified. In Figure 5(a), the most abundant families in all samples are Methanobacteriaceae (16%-75%), Methanoregulaceae (2%-74%), and Methanospirillaceae (3%-76%). According to the heat map at the general level (Figure 5(b)), five genera, i.e., *Methanolinea* (3%-74%), *Methanobacterium* (8%-73%), *Methanobrevibacter* (0-15%), *Methanospirillum* (0-21%), and *Methanothrix* (0-15%), can be considered as the core community displaying more than 90% abundance of each sample and confirming their key role in digesters (relative abundances of the archaea at the genus level are shown in Figure S4 of the Supplementary Materials). It is well known that *Methanolinea*, *Methanobacterium*, *Methanobrevibacter*, and *Methanospirillum* are the most common hydrogenotrophic methanogens [38, 44], for their ability to scavenge H_2_ by maintaining a low pH.

Among the archaeal community, *Methanothrix* (also called *Methanosaeta*) with a 100% similarity to *Methanothrix soehngenii* [45, 46] was the only acetoclastic methanogen detected (5.5% in R1 and 4.5% in R2 till VIII_a). Overall, these findings strongly confirmed the dominance of hydrogenotrophic methanogens among the archaeal community in both systems, indicating that the increasing H_2_ dosage is an effective way to promote the growth of hydrogenotrophic rather than acetoclastic methanogens.

Something worthy of note was that the hydrogenotrophic methanogens differed during the H_2_ dosage periods. More specifically, the most abundant genus *Methanolinea* was present in most samples except R1_VIII_c and R2_VIII_c. The NDMS results showed that most samples clustered together except these two samples. The second most abundant genus was *Methanobacterium*, *Methanobacterium palustre* which is known to utilize H_2_/CO_2_, for its growth and/or methane production [47]. Its abundance gradually increased with the increase of the H_2_/CO_2_ ratio demonstrating its role in the biogas upgrading.

## 4. Conclusions

The following conclusions can be drawn from the experimentation performed: (i) as already found with other studies [19], the stoichiometric H_2_/CO_2_ ratio (4 : 1) is not sufficient to complete the CO_2_ conversion to CH_4_ and achieve interesting low percentages of carbon dioxide in the biogas; this is also very likely affected by the H_2_ injection mode which determine the diffusion of the gas in the liquid phase. Further research is needed in order to optimize the hydrogen gas transfer process; (ii) by increasing the H_2_/CO_2_ ratio to 7 : 1, it was possible to efficiently maximize the CO_2_ conversion for the production of a biogas mainly composed of methane (max 90.3%); (iii) the sewage sludge degradation was not negatively affected by the incremental supplying of H_2_, while a significant alkalinity consumption was observed. In order not to impair the process, it is crucial to ensure that the organic influent is capable of reintegrating the alkalinity consumed; (iv) the efficiency of the process was ensured by the development of a specialized community, composed mainly of *Methanobacterium*, *Methanobrevibacter*, *Methanospirillum* (hydrogenotrophic methanogens), homoacetogens, hydrolytic, and acidogenic bacteria, thanks to the enrichment strategy; (v) during H_2_ periods, the two parallel reactors produced different methane rates but displayed similar trends. The results of this experiment are quite robust and repeatable, but further studies focused on this topic are needed in order to assess how the performance of the process, while introducing high concentrations of hydrogen, is susceptible to common small differences normally establishing in anaerobic digesters.

## Figures and Tables

**Table 1:** Operational parameters during experimental periods; in brackets, next to the period number, the H_2_/CO_2_ ratio adopted.

Period–H_2_/CO_2_	Duration (d)	Progressive days (begin-end)	OLR_SL_ (g COD·L^−1^·d^−1^)	OLR_tot_ (g COD·L^−1^·d^−1^)
I	97^(a)^	0-20	1.3 ± 0.6	1.3 ± 0.6
II−0.5	20	21-41	1.5 ± 0.2	1.5 ± 0.1
III–1	7	42-49	1.5 ± 0.1	1.6 ± 0.3
IV–2	8	50-58	1.6 ± 0	1.6 ± 0.4
V–3	8	59-67	1.6 ± 0	1.7 ± 0.2
VI–4	26	68-94	1.4 ± 0.2	1.7 ± 0.3
VII–6	12	95-107	1.5 ± 0.8	1.8 ± 0.8
VIII–7	33	108-141	1.5 ± 0.4	1.9 ± 0.6

^(a)^In all figures, only the last 20 days of Period I are shown.

**Table 2:** Chemical characterization (mean ± standard deviation) of inoculum and sludge mixture used.

Parameters	Sludge mixture	Inoculum
Total solids (TS) (gTS∙kg_FM_^−1^)^(a)^	26 ± 6	20 ± 2
Volatile solids (VS) (gVS∙kg_FM_^−1^)	18 ± 5	12 ± 3
VS/TS (%)	70	59 ± 1
COD_tot_ (gCOD∙kg_FM_^−1^)	12	5.5
TVFA (mgCOD∙L^−1^)	1254 ± 130	224
Alkalinity (mgCaCO_3_∙L^−1^)	1629 ± 261	5666 ± 148

^(a)^FM: fresh matter.

**Table 3:** Summary of reactors' gas mass flow (CO_2_, CH_4_, and H_2_) during the experimental periods.

Period–H_2_/CO_2_	CO_2_ (NmL∙d^−1^)		CH_4_ (NmL∙d^−1^)		H_2_ (NmL∙d^−1^)	
	R1	R2	R1	R2	R1	R2
I	788	894	2597	2772	0	0
II-0.5	955	1054	2813	3137	27	33
III–1	962	1034	2985	3319	42	28
IV–2	953	1094	3161	3612	43	38
V–3	1004	1307	3628	4404	54	65
VI–4	605	1147	3454	4542	203	349
VII–6	575	864	3597	4329	134	229
VIII–7	297	440	3353	4970	235	348

**Table 4:** Summary of reactor alkalinity and VS removal during the experimental periods.

Period	Reactor	Alkalinity (gCaCO_3_∙L^−1^)	VS removal (%)
I	R1	4.36	39%
	R2	4.33	37%
II	R1	4.04	44%
	R2	4.07	42%
III	R1	4.04	39%
	R2	4.01	40%
IV	R1	3.96	45%
	R2	4.02	47%
V	R1	3.99	41%
	R2	4.37	42%
VI	R1	3.36	35%
	R2	4.06	32%
VII	R1	3.48	39%
	R2	3.92	34%
VIII	R1	2.16	37%
	R2	3.63	32%

**Table 5:** Summary of literature studies of the in situ biogas upgrading technology associated with H_2_ injection.

Substrate	Reactor type	Reactor volume (L)	OLR (gVS∙L^−1^∙d^−1^)	T (°C)	pH	HRT (d)	H_2_ (LH_2_∙L^−1^∙d^−1^)	H_2_/CO_2_	H_2_ conversion (%)	CO_2_ removal (%)	CH_4_ (%)	Ref.
Potato starch	UASB	1.4	2.79	55	8.4	7	3.5	4 : 1	67	76	82	Bassani et al. [48]
Cattle manure	CSTR	0.6	1.85	55	8.3	14	1.8	4 : 1	>90	N/D	65	Luo et al. [13]
Cattle manure and cheese whey	CSTR	0.6	1.66	55	7.7-7.9	15	1.5-1.7	4 : 1	N/D	85	75	Luo and Angelidaki [49]
Cattle manure and cheese whey	CSTR	0.6	1.66	55	7.6-8.3	15	0.9-1.8	4 : 1	N/D	53-91	78-96	Luo and Angelidaki, [16]
Sewage sludge	CSTR	3	1,08	37	8.0	10	0.6-1.3	4 : 1	96	99	99	Wang et al. [33]
Sewage sludge	Batch	2	0.77	38	7.9-8.4	20	0.3-1.7	2 : 1-10 : 1	58-99	43-100	79-92	Agneessens et al. [19]
Sewage sludge	Semicontinuous	2.4	1-1.12	35	7.0-7.4	15	0, 0.62-0.2	1 : 1-4 : 1	96-100	13-49	80	Corbellini et al. [21]
Sewage sludge	CSTR	16	1.5	36.7	7.4	22	0.4-5.5	0-7 : 1	94-99	96.5	90.3	This study

CSTR: continuous stirred tank reactors; HRT: hydraulic retention time; OLR: organic loading rate; UASB: upflow anaerobic sludge blanket.

## Data Availability

All data used to support the findings of this study are available from the corresponding author upon request.

## Supplementary Materials

Supplementary Materials A Supplementary Materials file is provided, including: Table S1: Mann–Whitney test results for comparison between R1 and R2: significance value, total number of samples tested, and mean ranks calculated for the two reactors. Cases where null hypothesis is rejected are highlighted in grey. Table S2: Kruskal-Wallis test results for comparison between phases of reactors R1 and R2: significance value, total number of samples tested, and test statistic value. Cases where null hypothesis is rejected are highlighted in grey. Table S3: Dunn's pairwise test (Bonferroni correction) for multiple comparisons between phases of reactor R1 and R2: significance value. Cases where null hypothesis is rejected are highlighted in grey. Figure S1: methane yields during the start-up period without H_2_ addition, both for reactor R1 and R2. Table S4: read and amplicon sequence variants (ASV) distribution derived from the 16 samples in two parallel CSTR anaerobic digesters. Figure S2: rarefaction curves of the ASV obtained from 16S rRNA gene analysis of the bacterial community of R1 and R2 and (B) archaeal community in parallel anaerobic digestion reactors. Figure S3: relative abundance of the bacteria at the genus level of two parallel reactors at several sampling points. Figure S4: relative abundance of the archaea at the genus level of two parallel reactors at several sampling points.
