# Supplementary material for: Performance Analysis and Microbial Community Evolution of In Situ Biological Biogas Upgrading with Increasing H2/CO2 Ratio
Source: Archaea. 2021 Feb 9;2021:8894455. doi: 10.1155/2021/8894455 (PMC7889367; doi:10.1155/2021/8894455)
Supplement: Supplementary Materials — A Supplementary Materials file is provided, including: Table S1: Mann–Whitney test results for comparison between R1 and R2: significance value, total number of samples tested, and mean ranks calculated for the two reactors. Cases where null hypothesis is rejected are highlighted in grey. Table S2: Kruskal-Wallis test results for comparison between phases of reactors R1 and R2: significance value, total number of samples tested, and test statistic value. Cases where null hypothesis is rejected are highlighted in grey. Table S3: Dunn's pairwise test (Bonferroni correction) for multiple comparisons between phases of reactor R1 and R2: significance value. Cases where null hypothesis is rejected are highlighted in grey. Figure S1: methane yields during the start-up period without H2 addition, both for reactor R1 and R2. Table S4: read and amplicon sequence variants (ASV) distribution derived from the 16 samples in two parallel CSTR anaerobic digesters. Figure S2: rarefaction curves of the ASV obtained from 16S rRNA gene analysis of the bacterial community of R1 and R2 and (B) archaeal community in parallel anaerobic digestion reactors. Figure S3: relative abundance of the bacteria at the genus level of two parallel reactors at several sampling points. Figure S4: relative abundance of the archaea at the genus level of two parallel reactors at several sampling points. [file 8894455.f1.docx]

**Performance analysis and microbial community evolution of in-situ biological biogas upgrading with increasing H_2_/CO_2_ ratio**

Viola Corbellini^a*^, Cuijie Feng^a^, Micol Bellucci^a^, Arianna Catenacci^a^, Tatiana Stella^b^, Anna Espinoza-Tofalos^b^, Francesca Malpei^a^

**Supplementary information includes:**

Pages S1 to S9

**Table S1.** Mann-Whitney test results for comparison between R1 and R2: significance value, total number of samples tested, and mean ranks calculated for the two reactors. Cases where null hypothesis is rejected are highlighted in grey.

| **Test Type** | **Main Statistics** | **Tested variables** | | | | |
| --- | --- | --- | --- | --- | --- | --- |
|  |  | **Biogas rate** | **CO_2_ %** | **CH_4_ %** | **Alkalinity** | **TVFA** |
| **All Periods** | Significance | <0.001 | 0.252 | 0.097 | 0.054 | 0.352 |
|  | Total N | 284 | 100 | 100 | 86 | 60 |
|  | Mean Rank - R1 | 103 | 47.2 | 55.3 | 38.3 | 32.6 |
|  | Meank Rank - R2 | 182 | 53.8 | 45.7 | 48.7 | 28.4 |
| **Period I** | Significance | 0.019 | 0.021 | 0.021 | 0.805 | 1.000 |
|  | Total N | 42 | 28 | 28 | 14 | 4 |
|  | Mean Rank - R1 | 17.05 | 10.93 | 18.07 | 7.86 | 2.50 |
|  | Meank Rank - R2 | 25.95 | 18.07 | 10.93 | 7.14 | 2.50 |
| **Period II** | Significance | <0.001 | 0.436 | 0.489 | 0.853 | 0.151 |
|  | Total N | 42 | 18 | 18 | 20 | 10 |
|  | Mean Rank - R1 | 14.57 | 10.56 | 8.56 | 18.8 | 7 |
|  | Meank Rank - R2 | 28.43 | 8.44 | 10.44 | 10.2 | 4 |
| **Period III** | Significance | <0.001 | 0.400 | 0.100 | 1.000 | 1.000 |
|  | Total N | 16 | 6 | 6 | 6 | 4 |
|  | Mean Rank - R1 | 4.5 | 4.33 | 2 | 3.33 | 2.5 |
|  | Meank Rank - R2 | 12.5 | 2.67 | 5 | 3.67 | 2.5 |
| **Period IV** | Significance | 0.001 | 1.000 | 0.400 | 1.000 | 1.000 |
|  | Total N | 18 | 6 | 6 | 5 | 6 |
|  | Mean Rank - R1 | 5.67 | 3.33 | 2.67 | 3.00 | 3.33 |
|  | Meank Rank - R2 | 13.33 | 3.67 | 4.33 | 3.00 | 3.67 |
| **Period V** | Significance | <0.001 | 0.100 | 0.100 | 0.200 | 0.667 |
|  | Total N | 18 | 6 | 6 | 6 | 4 |
|  | Mean Rank - R1 | 5.00 | 2.00 | 5.00 | 2.33 | 3.00 |
|  | Meank Rank - R2 | 14.00 | 5.00 | 2.00 | 4.67 | 2.00 |
| **Period VI** | Significance | <0.001 | 0.008 | 0.008 | 0.004 | 0.690 |
|  | Total N | 54 | 10 | 10 | 11 | 10 |
|  | Mean Rank - R1 | 14.74 | 3.00 | 8.00 | 3.00 | 6.00 |
|  | Meank Rank - R2 | 40.26 | 8.00 | 3.00 | 8.50 | 5.00 |
| **Period VII** | Significance | 0.003 | 0.200 | 0.100 | 0.100 | 1.000 |
|  | Total N | 26 | 6 | 6 | 6 | 6 |
|  | Mean Rank - R1 | 9.23 | 2.33 | 5.00 | 2.00 | 3.67 |
|  | Meank Rank - R2 | 17.77 | 4.67 | 2.00 | 5.00 | 3.33 |
| **Period VIII** | Significance | <0.001 | 0.315 | 0.230 | 0.004 | 0.798 |
|  | Total N | 68 | 20 | 20 | 18 | 16 |
|  | Mean Rank - R1 | 22.41 | 9.1 | 12.6 | 6 | 8.88 |
|  | Meank Rank - R2 | 46.59 | 11.9 | 8.4 | 13 | 8.12 |

**Table S2.** Kruskal-Wallis test results for comparison between phases of reactors R1 and R2: significance value, total number of samples tested and test statistic value. Cases where null hypothesis is rejected are highlighted in grey.

| **Reactor** | **Main Statistics** | **Tested variables** | | |
| --- | --- | --- | --- | --- |
|  |  | **Biogas rate** | **CO_2_ %** | **CH_4_ %** |
| **R1** | Significance | <0.001 | <0.001 | <0.001 |
|  | Total N | 142 | 50 | 50 |
|  | Test Statistic | 103 | 44.6 | 44.6 |
| **R2** | Significance | <0.001 | <0.001 | <0.001 |
|  | Total N | 142 | 50 | 50 |
|  | Test Statistic | 98.7 | 43.2 | 36.8 |

**Table S3.** Dunn’s pairwise test (Bonferroni correction) for multiple comparisons between phases of reactor R1 and R2: significance value. Cases where null hypothesis is rejected are highlighted in grey.

| **Period →** | | **I** | | | **II** | | | | **III** | | | | **IV** | | | | **V** | | | | **VI** | | | | **VII** | | | | **VIII** | | |
| --- | --- | --- | --- | --- | --- | --- | --- | --- | --- | --- | --- | --- | --- | --- | --- | --- | --- | --- | --- | --- | --- | --- | --- | --- | --- | --- | --- | --- | --- | --- | --- |
| **Reactor →** | | **R1** | **R2** | | **R1** | | **R2** | | **R1** | | **R2** | | **R1** | | **R2** | | **R1** | | **R2** | | **R1** | | **R2** | | **R1** | | **R2** | | **R1** | | **R2** |
| **Variable →** | | BIOGAS | | | | | | | | | | | | | | | | | | | | | | | | | | | | | |
| **Period** | **I** | - | | - | | - | | - | | - | | - | | - | | - | | - | | - | | - | | - | | - | | - | | - | - |
|  | **II** | 1 | | 1 | | - | | - | | - | | - | | - | | - | | - | | - | | - | | - | | - | | - | | - | - |
|  | **III** | 1 | | 1 | | 1 | | 1 | | - | | - | | - | | - | | - | | - | | - | | - | | - | | - | | - | - |
|  | **IV** | 0.18 | | 0.204 | | 1 | | 1 | | 1 | | 1 | | - | | - | | - | | - | | - | | - | | - | | - | | - | - |
|  | **V** | <0.001 | | <0.001 | | 0.075 | | 0.005 | | 1 | | 0.226 | | 1 | | 1 | | - | | - | | - | | - | | - | | - | | - | - |
|  | **VI** | 0.006 | | <0.001 | | 1 | | <0.001 | | 1 | | 0.004 | | 1 | | 0.049 | | 1 | | 1 | | - | | - | | - | | - | | - | - |
|  | **VII** | 0.109 | | <0.001 | | 1 | | 0.026 | | 1 | | 0.945 | | 1 | | 1 | | 1 | | 1 | | 1 | | 1 | | - | | - | | - | - |
|  | **VIII** | 0.878 | | <0.001 | | 1 | | <0.001 | | 1 | | 0.041 | | 1 | | 0.419 | | 0.054 | | 1 | | 1 | | 1 | | 1 | | 1 | | - | - |
| **Variable →** | | % CO_2_ | | | | | | | | | | | | | | | | | | | | | | | | | | | | | |
| **Period** | **I** | - | | - | | - | | - | | - | | - | | - | | - | | - | | - | | - | | - | | - | | - | | - | - |
|  | **II** | 0.415 | | 1 | | - | | - | | - | | - | | - | | - | | - | | - | | - | | - | | - | | - | | - | - |
|  | **III** | 1 | | 1 | | 1 | | 1 | | - | | - | | - | | - | | - | | - | | - | | - | | - | | - | | - | - |
|  | **IV** | 1 | | 1 | | 1 | | 1 | | 1 | | 1 | | - | | - | | - | | - | | - | | - | | - | | - | | - | - |
|  | **V** | 1 | | 1 | | 0.265 | | 0.701 | | 1 | | 1 | | 1 | | 1 | | - | | - | | - | | - | | - | | - | | - | - |
|  | **VI** | 1 | | 0.2 | | 0.004 | | 0.043 | | 1 | | 1 | | 1 | | 1 | | 1 | | 1 | | - | | - | | - | | - | | - | - |
|  | **VII** | 1 | | 0.191 | | 0.018 | | 0.048 | | 1 | | 1 | | 1 | | 1 | | 1 | | 1 | | 1 | | 1 | | - | | - | | - | - |
|  | **VIII** | 0.001 | | <0.001 | | <0.001 | | <0.001 | | 0.06 | | 0.386 | | 0.249 | | 1 | | 1 | | 1 | | 1 | | 1 | | 1 | | 1 | | - | - |
| **Variable →** | | % CH_4_ | | | | | | | | | | | | | | | | | | | | | | | | | | | | | |
| **Period** | **I** | - | | - | | - | | - | | - | | - | | - | | - | | - | | - | | - | | - | | - | | - | | - | - |
|  | **II** | 0.209 | | 1 | | - | | - | | - | | - | | - | | - | | - | | - | | - | | - | | - | | - | | - | - |
|  | **III** | 1 | | 1 | | 1 | | 1 | | - | | - | | - | | - | | - | | - | | - | | - | | - | | - | | - | - |
|  | **IV** | 1 | | 1 | | 1 | | 1 | | 1 | | 1 | | - | | - | | - | | - | | - | | - | | - | | - | | - | - |
|  | **V** | 1 | | 1 | | 1 | | 0.86 | | 1 | | 1 | | 1 | | 1 | | - | | - | | - | | - | | - | | - | | - | - |
|  | **VI** | 1 | | 1 | | 0.008 | | 1 | | 0.624 | | 1 | | 1 | | 1 | | 1 | | 1 | | - | | - | | - | | - | | - | - |
|  | **VII** | 1 | | 1 | | 0.013 | | 0.031 | | 0.415 | | 1 | | 1 | | 1 | | 1 | | 1 | | 1 | | 0.902 | | - | | - | | - | - |
|  | **VIII** | 0.004 | | 0.002 | | <0.001 | | <0.001 | | 0.009 | | 1 | | 0.191 | | 1 | | 1 | | 1 | | 1 | | 0.013 | | 1 | | 1 | | - | - |


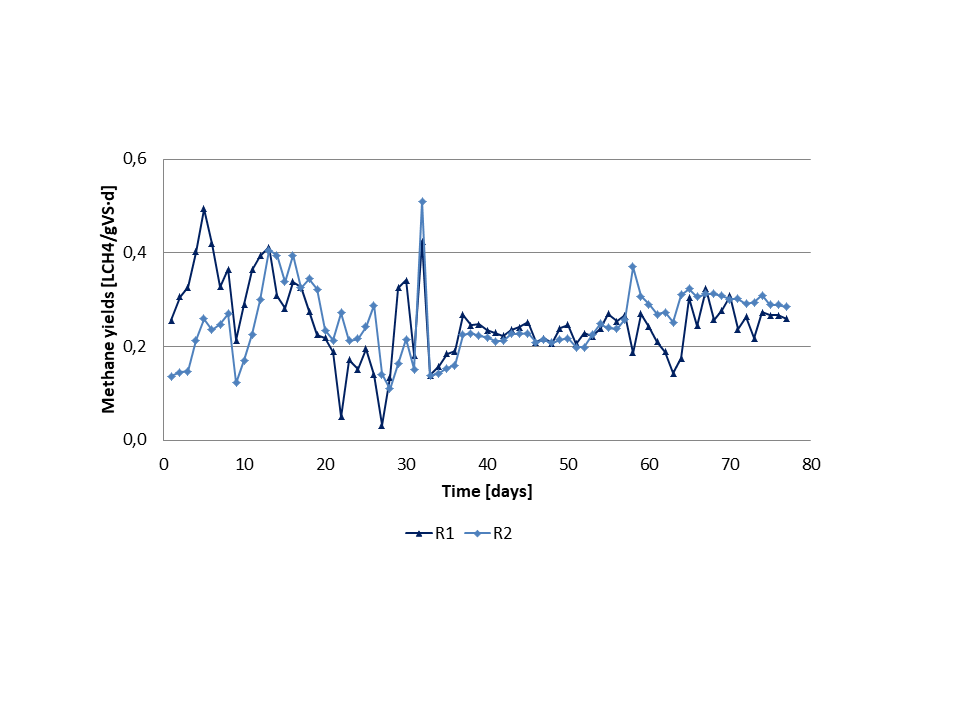


**Figure S1.** Methane yields during the start-up period without H_2_ addition, both for reactor R1 and R2.

**Table S4.** Read and amplicon sequence variants (ASV) distribution derived from the 16 samples in two parallel CSTR anaerobic digesters.

| **Sample ID** | **Bacteria** | |  | **Archaea** | |
| --- | --- | --- | --- | --- | --- |
|  | **ASV** | **Sequence** |  | **ASV** | **Sequences** |
| R1-IV | 680 | 37771 |  | 54 | 70305 |
| R1-V | 653 | 38793 |  | 53 | 26490 |
| R1-VI_a | 798 | 61892 |  | 45 | 35968 |
| R1-VI_b | 542 | 33570 |  | 47 | 27231 |
| R1-VII | 540 | 36197 |  | 54 | 38404 |
| R1-VIII_a | 208 | 6049 |  | 58 | 37199 |
| R1-VIII_b | 644 | 47271 |  | 77 | 63920 |
| R1-VIII_c | 490 | 23885 |  | 5 | 227 |
| R2-IV | 279 | 11730 |  | 30 | 17385 |
| R2-V | 794 | 63517 |  | 63 | 56080 |
| R12-VI_a | 743 | 49881 |  | 59 | 25406 |
| R2-VI_b | 554 | 33050 |  | 52 | 32938 |
| R2-VII | 374 | 17659 |  | 70 | 40254 |
| R2-VIII_a | 440 | 21123 |  | 43 | 14328 |
| R2-VIII_b | 421 | 20819 |  | 5 | 201 |
| R2-VIII_c | 263 | 11088 |  | 33 | 9647 |

**
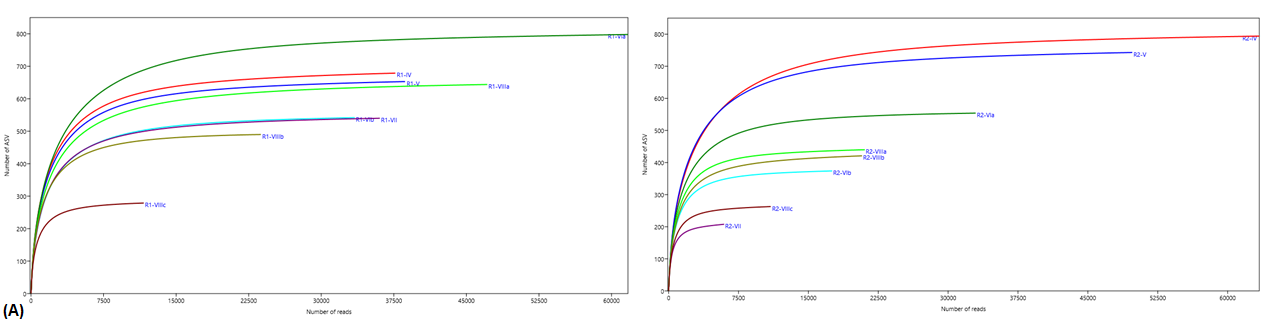
**

**
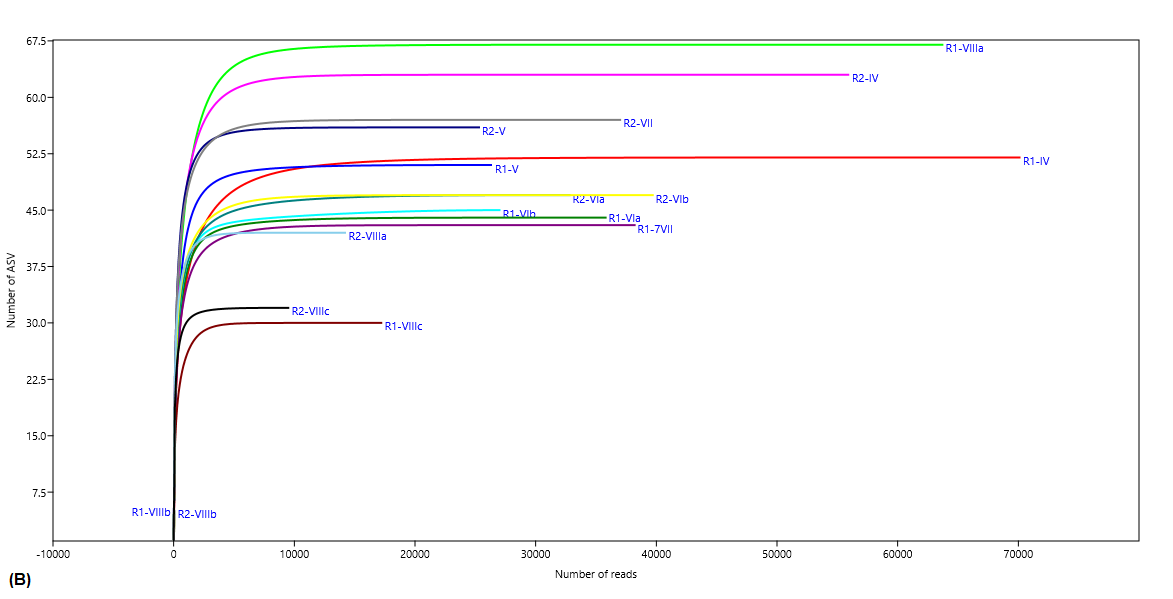
**

**Figure S2.** Rarefaction curves of the ASV obtained from 16S rRNA gene analysis of the bacterial community of R1 and R2 and (B) archaeal community in parallel anaerobic digestion reactors.

Figure S3. Relative abundance of the bacteria at the genus level of two parallel reactors at several sampling points.

Figure S4. Relative abundance of the archaea at the genus level of two parallel reactors at several sampling points.
